# A Rare Case of Parotid Stone Adherence to the Facial Nerve

**DOI:** 10.7759/cureus.57614

**Published:** 2024-04-04

**Authors:** Majid Althobaiti, Nader S Alharbi, Abdullah Alhajlah, Faisal A Althwiny, Muhammad K Amin

**Affiliations:** 1 Department of Otolaryngology - Head and Neck Surgery, Security Forces Hospital, Riyadh, SAU; 2 Department of Otolaryngology, Shaqra University, Riyadh, SAU; 3 Department of Otolaryngology, Imam Mohammad Ibn Saud Islamic University, Riyadh, SAU

**Keywords:** rare finding, sialadenitis, surgical challenges, sialendoscopy, facial nerve, combined endoscopic transcutaneous approach, parotid gland sialolithiasis

## Abstract

This case report details a complex case of parotid gland sialolithiasis with stones adherent to the facial nerve, a scenario that presents a significant surgical challenge. Traditional sialendoscopy failed to address the condition in a 23-year-old female patient, leading to the adoption of a combined endoscopic transcutaneous approach. This method successfully resolved the condition without intraoperative complications, maintaining intact facial nerve function postoperatively. The case emphasizes the importance of individualized surgical strategy and expert technique in advanced parotid surgery, advocating this approach for similarly complex sialolithiasis cases.

## Introduction

The largest salivary gland, the parotid, can occasionally develop diseases like sialolithiasis, which is characterized by the development of calcified stones in the salivary ductal system. The majority of cases of recurrent sialadenitis are caused by sialolithiasis, which may lead to severe morbidity because it causes pain and swelling in the affected gland [1]. While a lesser percentage of these stones are observed in the parotid gland, the submandibular gland is where the majority of them originate [2,3].

Conventional approaches to managing parotid stones involve the implementation of conservative measures, including sialogogues and hydration to promote salivation, as well as minimally invasive procedures like sialendoscopy [1]. In cases of sialolithiasis, sialendoscopy is used for both diagnostic and therapeutic purposes. This technique makes it relatively simple to remove stones using an endoluminal approach, which has a high success rate and low morbidity, particularly when the stones are inside the ductal system [4,5]. In spite of the great success rate of minimally invasive procedures, managing certain instances is still challenging, especially in cases where the stones are impacted and cannot be removed with oral methods alone [6].

The combined endoscopic and transcutaneous method is a useful substitute in situations where conservative or minimally invasive treatments are insufficient [6,7,8]. Using endoscopic transillumination and ultrasonographic guidance, this approach locates the stone precisely so that it can be removed with a well-placed transcutaneous incision. The high success rates of this approach have been frequently reported in the literature [9,10,11].

This case study illustrates the first reported case of a parotid stone attaching to the facial nerve, an unusual presentation that may present surgeons with unexpected challenges. Highlighting such a novel finding emphasizes the complexities of treating parotid gland sialolithiasis and the significance of a precise surgical plan to ensure the patient's outcome. By sharing our unique experience, we hope to contribute to a broader understanding of the possible complications of parotid surgery.

## Case presentation

A 23-year-old female with no known chronic diseases and no family history of similar conditions presented to our department with a three-year history of recurrent, painful swelling in the right parotid gland. The patient described multiple episodes of acute pain and swelling that typically worsened with meals.

Previously, the patient had sought treatment for what was diagnosed as recurrent sialadenitis due to right parotid gland sialolithiasis and had undergone an unsuccessful sialendoscopy at another hospital. Dissatisfied with the persistent symptoms and lack of improvement, she approached our facility for a second opinion and further intervention.

On physical examination, there was noticeable enlargement of the right parotid gland, with tenderness palpable over the affected area. There were no signs of facial weakness, and the rest of the head and neck examination, including the oral cavity, oropharynx, and neck, was unremarkable. The patient did not report any xerostomia, fever, chills, or weight loss. Additionally, there was no history of smoking, alcohol use, or radiation exposure.

**Figure 1:**
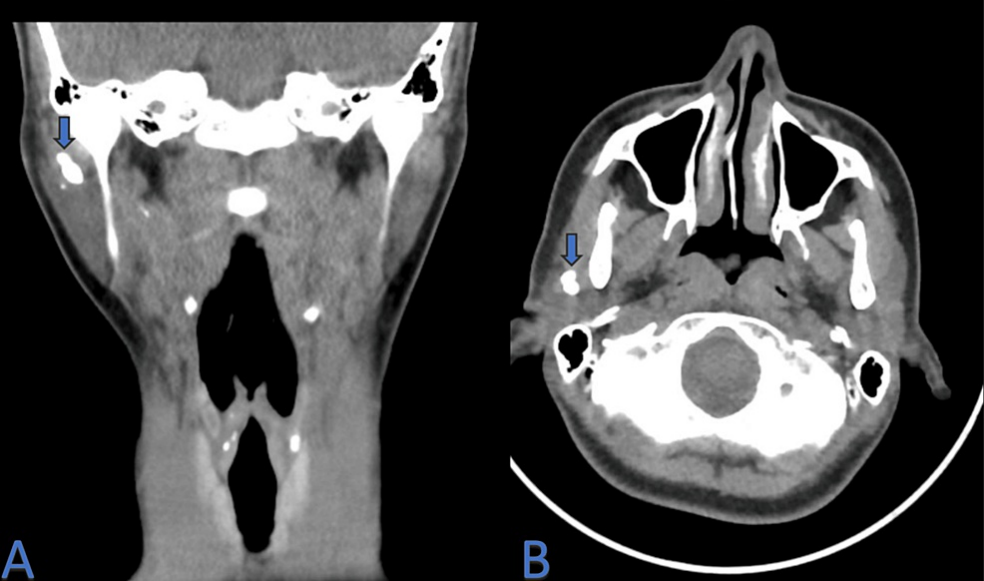
Non-contrasted neck CT scan Arrows on coronal (A) and axial (B) views of a non-contrasted neck CT scan pinpoint multiple intrasubstance radiopaque stones in the right parotid gland, with the largest measuring approximately 0.6 x 0.5 cm. CT: computed tomography

A review of a CT scan previously performed at another institution showed intraductal and intraglandular stones within the right parotid gland (Figure 1). No other abnormalities, such as masses or significant lymphadenopathy, were identified on the scan, and the left parotid gland appeared normal.

**Figure 2:**
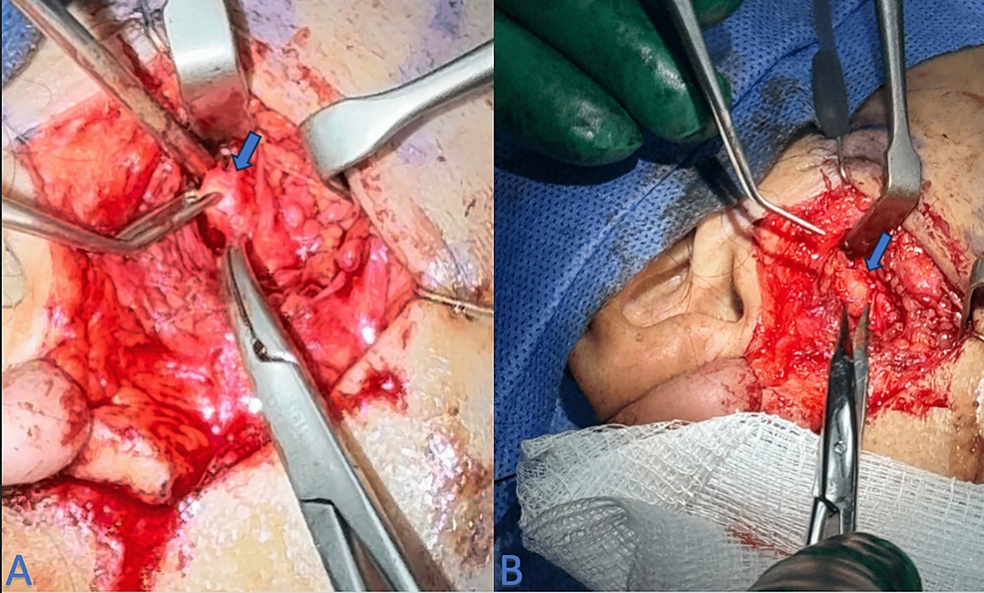
Parotid sialolith adherent to the facial nerve (arrow) The intraoperative image shows an unusual parotid stone firmly attached to the facial nerve.

Given the patient's history of recurrent symptoms and the imaging findings, we considered a combined endoscopic and transcutaneous approach to manage her condition. Intraoperatively, we encountered multiple stones, the largest of which was found to be unusually adherent to the facial nerve (Figure 2). This presented a unique challenge, as the proximity to the nerve increased the risk of iatrogenic injury.

**Figure 3:**
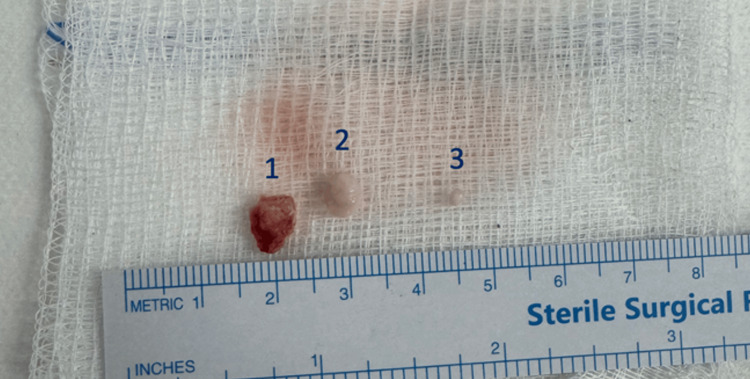
Variably shaped parotid stones Three distinct parotid sialoliths of varying sizes and shapes were successfully extracted from the parotid duct without complications or nerve injury.

A cautious dissection was performed to delicately free the stone from the facial nerve. We successfully extracted all stones without any intraoperative complications, and the integrity of the facial nerve was preserved (Figure 3).

Postoperatively, the patient's recovery was uneventful. During the three-month follow-up period, she remained asymptomatic with no recurrence of swelling or pain. The physical examination was consistently unremarkable, with maintained facial nerve function and no signs of infection or other complications. These follow-up findings were highly encouraging, highlighting the success of the surgical intervention and the resolution of the patient's chronic condition.

## Discussion

The management of parotid gland sialolithiasis necessitates a multidisciplinary approach, particularly when faced with anatomical variations that impede standard treatments. The case presented emphasizes a landmark finding: the first reported instance of a parotid stone attached directly to the facial nerve. This unprecedented surgical challenge required tailored treatment planning, employing the combined endoscopic transcutaneous approach for the removal of parotid stones.

The progress of parotid stone management represents a shift towards less invasive surgical techniques and more gland-preserving procedures. Historically, parotidectomy was the standard treatment, but the risk of facial nerve damage and the need to preserve gland function have resulted in the development of alternate methods [8]. The advent of sialendoscopy has become a pivotal change in the management of small (<4 mm) non-impacted stones, offering a less invasive option with a focus on gland preservation [8].

Treatment strategies for parotid gland sialolithiasis tend to be more complex, especially when the stones are larger than 4 mm or impacted [8]. One of the primary options is to fragment the large stone into smaller pieces that can be more easily removed. This fragmentation can be accomplished by extracorporeal shock wave lithotripsy (ESWL) or endoluminal laser fragmentation. ESWL has a reported success rate of about 60% for completely eliminating stones [8]. Furthermore, in some circumstances, ESWL can be efficiently paired with sialendoscopy procedures to remove stone pieces following fragmentation [12,13]. However, despite the use of sialendoscopy and ESWL, an estimated 10% of sialoliths are still resistant to endoscopic removal [8]. These persistent stones can cause frequent glandular inflammation and swelling, posing an ongoing dilemma for patients and physicians.

Another treatment option is to utilize a laser, such as thulium:YAG laser, to disintegrate the stones. According to the literature, this approach achieves an 80% success rate [8]. Despite this, the need for expensive equipment and specific training for these procedures may limit their widespread availability, making them less accessible in particular clinical settings [8,14].

The described combined endoscopic and transcutaneous approach utilized in our case is an alternative option in case the above-mentioned techniques are not available or have previously failed. It is a well-established alternative with a documented high success rate [9,10,11]. To the best of our knowledge, our hospital is the only center in the Gulf countries offering this particular procedure. The success of this procedure is greatly influenced by the surgeon's expertise in this technique and choice of it for the indicated cases. As per Nahlieli et al., the criteria for employing a combined approach are the presence of large parotid stones exceeding 4 mm, impacted stones, or cases where an endoscopic removal has previously been unsuccessful, as in the case presented [6].

The combined endoscopic and transcutaneous approach has several advantages. It offers a minimally invasive solution that can be highly effective for stones that are not amenable to endoscopic techniques alone [11]. This approach may also reduce the need for more extensive surgical procedures, which carry higher risks of complications and longer recovery times. Furthermore, it allows for the preservation of salivary gland function and has a lower likelihood of causing aesthetic deficits compared to traditional open approaches [9].

However, this technique is not without its limitations. One of the inherent risks of this procedure is the potential for injury to the buccal branch of the facial nerve, which can lead to temporary or permanent facial paralysis [6]. The requirement for specialized equipment and the necessity for a surgeon who is an expert in the technique can also present significant limitations, as these resources may not be readily available in all surgical centers [9].

Our significant discovery of the parotid stone's direct attachment to the facial nerve necessitated a highly precise dissection, with magnification and nerve monitoring imperative to avoid iatrogenic injury. The successful preservation of nerve function postoperatively, with no resulting deficits, highlights the critical nature of our approach in such rare and complex sialolithiasis cases. This case reinforces the literature on the importance of cautious surgical planning and intraoperative decision-making and may guide future surgical strategies when encountering similar scenarios.

## Conclusions

The presented case of a 23-year-old female with parotid gland sialolithiasis was notably challenging due to the adherence of a large stone to the facial nerve, which required a cautious dissection to avoid nerve damage and was managed by a combined approach. The favorable postoperative outcome, characterized by an absence of complications and intact facial nerve function, reinforces the combined approach as a viable and effective alternative to traditional techniques. This case may thereby contribute a valuable addition to the existing literature on the management of sialolithiasis, emphasizing the importance of choosing the right surgical technique for the right case. Our experience affirms the effectiveness of the combined endoscopic and transcutaneous approach, particularly in cases where stones are adherent to critical structures and traditional methods are inadequate or have previously failed.
